# Stratified Management for Bacterial Infections in Late Preterm and Term Neonates: Current Strategies and Future Opportunities Toward Precision Medicine

**DOI:** 10.3389/fped.2021.590969

**Published:** 2021-04-01

**Authors:** Fleur M. Keij, Niek B. Achten, Gerdien A. Tramper-Stranders, Karel Allegaert, Annemarie M. C. van Rossum, Irwin K. M. Reiss, René F. Kornelisse

**Affiliations:** ^1^Division of Neonatology, Department of Pediatrics, Erasmus Medical Center-Sophia Children's Hospital, Rotterdam, Netherlands; ^2^Department of Pediatrics, Franciscus Gasthuis and Vlietland, Rotterdam, Netherlands; ^3^Department of Development and Regeneration, Department of Pharmaceutical and Pharmacological Sciences, Katholieke Universiteit Leuven, Leuven, Belgium; ^4^Department of Clinical Pharmacy, Erasmus Medical Center Rotterdam, Rotterdam, Netherlands; ^5^Division of Infectious Diseases, Department of Pediatrics, Erasmus Medical Center-Sophia Children's Hospital, Rotterdam, Netherlands

**Keywords:** neonatal bacterial infection, diagnostics, antibiotic stewardship, precision medicine, late preterm and term neonates

## Abstract

Bacterial infections remain a major cause of morbidity and mortality in the neonatal period. Therefore, many neonates, including late preterm and term neonates, are exposed to antibiotics in the first weeks of life. Data on the importance of inter-individual differences and disease signatures are accumulating. Differences that may potentially influence treatment requirement and success rate. However, currently, many neonates are treated following a “one size fits all” approach, based on general protocols and standard antibiotic treatment regimens. Precision medicine has emerged in the last years and is perceived as a new, holistic, way of stratifying patients based on large-scale data including patient characteristics and disease specific features. Specific to sepsis, differences in disease susceptibility, disease severity, immune response and pharmacokinetics and -dynamics can be used for the development of treatment algorithms helping clinicians decide when and how to treat a specific patient or a specific subpopulation. In this review, we highlight the current and future developments that could allow transition to a more precise manner of antibiotic treatment in late preterm and term neonates, and propose a research agenda toward precision medicine for neonatal bacterial infections.

## Background

### Neonatal Bacterial Sepsis and Infections

Bacterial infection can lead to sepsis, a state in which dysregulation of the hosts' response to the infection leads to potentially fatal organ dysfunction (1). Consensus on specific criteria to define this state in neonates is still lacking, and hitherto the most common proxy definition of neonatal sepsis is the presence of a positive blood culture (indicating bacteremia), or a positive cerebrospinal fluid culture (indicating meningitis). This paper will acknowledge the limitations of this proxy definition by incorporating the uncertainties it carries when making clinical decisions, demonstrating how precision medicine can help with those decisions and highlighting how a future consensus definition can further advance precision medicine in treating neonatal sepsis (2, 3).

Neonatal bacterial infections (bacterial infections presenting in the first 28 days of life) affect an estimated 3.0 million neonates yearly, resulting in significant morbidity and mortality (4–6). Early-onset sepsis (EOS), defined as bacteremia within the first 72 h after birth, affects an average of 10 per 1,000 live births among neonates born below 33 weeks of gestation. Lower incidences of 0.73 and 0.56 per 1,000 live births are seen among late preterm neonates [gestational age (GA): 34–36 weeks] and term neonates (GA ≥ 37 weeks), respectively (7). Although the incidence and mortality remain much higher among extreme preterm neonates, the absolute number of cases of EOS is higher among late preterm- and term neonates, since prematurity (GA < 37 weeks) affects about 11% of total live births of which 85% occurs in the late preterm period (GA 32–37 weeks) (8). Pathogens associated with EOS include both Gram-positive and -negative pathogens with significantly higher rates of Gram-negatives, especially *Escherichia coli* (*E. coli*) infections among preterm neonates compared to term neonates. In contrast, Group B *Streptococcus* (GBS) infections seem to affect term neonates more frequently (7). Late-onset sepsis (LOS) involves infections occurring >72 h after birth. However, the onset of late-onset GBS infection is frequently defined in literature as an infection that occurs >7 days after birth (9). LOS develops due to contact of the host with environmental organisms and includes both hospital-acquired infections (nosocomial infections) and community-acquired infections. Causative pathogens include skin commensals such as coagulase-negative staphylococci (CoNS) and *Staphylococcus aureus*, and gut-associated microbiota such as *E. coli*. The latter is thought to reach the bloodstream through translocation across the immature intestine (10). CoNS can be pathogenic, especially for preterm neonates, and these infections are therefore often seen in hospitalized preterm neonates undergoing invasive procedures or with intravenous catheters (5, 11).

### Host Susceptibility

The neonatal immune system is a complex network, constantly adapting and undergoing an age-dependent maturation during gestation and after birth. It is shaped by intra- and extra uterine exposures such as antigens, medication, and environmental factors, necessitating both immunotolerance (to prevent immunoreactivity between mother and fetus) and pro-inflammation (infection protection) (12). Reviewing the neonatal immune system and the development of neonatal sepsis is beyond the scope of this review. Several excellent reviews have been published elaborating on this topic (13–15). In summary, in the presence of a pathogen, the host' immune system dysregulates; alternating phases of hyper inflammation (“cytokine storm”), potentially causing multi-organ failure, and immunosuppression (window for opportunistic infections). As pathogen exposure *in utero* is limited, and thus memory function is lacking, neonates primary rely on innate immunity and maternal transplacental immunoglobulin G (IgG) in early life (13).

Consequently, neonates are vulnerable for infections. The differences between preterm and term neonatal immune development do partly explain the differences observed in infection and sepsis incidence and severity between both groups. However, this does not explain interpatient variability in infection susceptibility seen within each group. Many late preterm and term neonates, fortunately, develop only mild symptoms when exposed to a pathogen. But a small group of late preterm and term infants, without any apparent co-morbidities, does develop severe infection (16).

### Balancing Under- and Over-Treatment

In case of a clinical suspicion of neonatal bacterial infection, empirical therapy using intravenous administration of broad-spectrum antibiotics is generally started without further delay. Although lifesaving in case of a true infection, unnecessary and inadequate antibiotic use has many downsides for both patient and health care system including gut microbiome alterations, multi-drug resistance and costs (17, 18). The balance between timely and proper diagnosis and overtreatment of neonatal infections remains a daily clinical challenge. This is illustrated by the fact that, in most cases, antibiotics can be discontinued after 36–48 h when clinical and laboratory signs are reassuring, or are continued in the presence of clinical and laboratory signs of infection, despite culture negativity (culture-negative infection) (19, 20). As a result, a substantial number of late preterm- and term neonates are exposed to intravenous antibiotics in their first weeks of life and antibiotics are among the most prescribed drugs on the neonatal medium and intensive care units (NICU) (11). Wide variation in neonatal antibiotic exposure between countries and hospitals, unexplained by infection rates, demonstrates the difficulty in ascertaining neonatal infection This, together with the differences in susceptibility, highlights the need for precision medicine in neonatal sepsis (21, 22).

### Precision Medicine

The term precision medicine has emerged in the last years. Yet, no consensus definition exists and many other terms such as “personalized” or “stratified” medicine are used interchangeably. However, experts view precision medicine as a novel, improved concept that goes beyond the personal doctor-patient relation (23). Precision medicine is viewed as a way to identify, stratify, and treat patients using large-scale data that relate to the underlying causes of their disease (24). It implicates deep phenotyping of patients in which information is gathered at different levels (“big data”) and involves the use of clinical- and life style data, omics and biomarkers. Collected data are used for the development of algorithms and models for disease or therapy risk assessment, screening, diagnosis, treatment selection, prognosis, prevention, and surveillance or monitoring. Those tools allow a more tailored and targeted therapy (24). Precision medicine is not yet widely practiced in the field of neonatal sepsis. However, recent studies evaluating different tools for neonatal sepsis have been performed and results could be a step toward better understanding of the disease-specific pathophysiology (25).

This could be achieved through the use of newer techniques, such as “*omics*,” in addition to conventional methods. The suffix -*omics* generally refers to the biotechnology that characterizes and quantifies biological molecules and structures at different levels of an organism. It compromises *genomics, transcriptomics, proteomics*, and *metabolomics* and allows detection of a “unique barcode” that could predict the underlying response to infection for an individual patient (26). We will discuss some of the current “omics” findings applicable to late preterm and term neonates.

#### Genomics

Variation in the host genetics could partly explain the variability in disease susceptibility. Genetic signatures or polymorphisms have been discovered for several infectious diseases. One of the well-known examples is malaria, where patients suffering from haemoglobinopathies are protected against malaria because of an altered erythrocyte structure (27). Another example, applicable to the pediatric population, is the PAI-1 polymorphism in meningococcal sepsis. PAI-1 is an acute phase protein and elevated concentrations correlate with disease severity and mortality. The 4G/4G PAI-1 polymorphism is associated with higher concentrations of PAI-1, and thus worse prognosis, compared to other genotypes (28). Sex has also been found to influence sepsis susceptibility and outcome as reflected in the increased vulnerability to infections and higher sepsis mortality in male neonates compared to females. The X-chromosome encodes multiple genes related to the immune system. Moreover, it is involved in the generation of the sex hormones, of which estrogen has showed to influence several pathways of innate immunity, possibly explaining a better sepsis outcome in females compared to males (29–31). Finally, pharmacogenomics allows us to study genetic polymorphisms associated with pharmacokinetics or –dynamics (drug response).

#### Transcriptomics

Transcriptomics refers to the study of the ribonucleic acid (RNA) transcripts allowing to study changes in gene expression over time or under certain circumstances, such as sepsis. Studies using next-generation sequencing and RNA-sequencing have showed that differences are present in gene expression between septic and non-septic neonates with overexpression of genes related to innate immunity and inflammation [CD177; Matrix metalloproteinase-8 (MMP8); tumor necrosis factor-alpha (TNF-α)] (25, 32). Moreover, Cernada et al. reported that genome wide expression profiles differ between Gram-negative and Gram-positive sepsis (32). A recent published study by Ng et al. reported whole blood transcriptomic profiles of very preterm infants (*n* = 18) with proven, possible, and no sepsis. Significant differences were seen in gene expression between proven and no sepsis cases. Altered genes were associated with cytokine signaling, pattern recognition and metabolism (33). With regard to gestational age, Cernada et al. (32) reported no differences in gene expression between very preterm- and late-preterm and term neonates. However, the proportion of late preterm and term neonates included in the study is low, and those are, in most cases, suspected sepsis episodes, not culture proven sepsis. Wynn et al. (34) reported significant differences between the transcriptome of septic neonates and that of septic infants and older children, illustrating an association with developmental age (35).

#### Proteomics

Protein-coding genes eventually lead to the expression of specific proteins, and the structure, function and interaction of those proteins can be studied revealing potentially useful biomarkers for neonatal sepsis. One of the advantages of proteomics is that these new biomarkers can be discovered through a hypothesis-free approach as more than a thousand proteins and modifications can be screened using mass spectrometry (25).

#### Metabolomics

The metabolome includes all low molecular weight molecules produced by the human body and is considered to be a reflection of a patient's phenotype and real-time physiological condition (36). Metabolomic perturbations due to a higher energy demand and oxidative stress during sepsis can therefore be used as possible predictors or biomarkers for neonatal sepsis (37, 38). Only a few studies have used metabolomics in neonatal sepsis revealing different metabolic pathways involved in neonatal sepsis. Levels of metabolites of energy and glucose metabolism (glucose, glutamine, and lactate) were significant altered in septic neonates (39, 40).

#### Microbiome

The microbial community of the gut (“gut microbiome”) is shown to be an important influencer of health and disease. It protects from potential pathogens through both the development of barrier function and by shaping the immunological and metabolic pathways. Alterations in early life have been associated with several diseases, such as asthma and obesity, at a later age (41, 42). Significant differences are seen between the gut microbiome of preterm neonates compared to that of term neonates, independent of other environmental factors (“exposome”) that influence bacterial colonization such as the mode of delivery, type of feeding or the administration of antibiotics (43). Decreased bacterial diversity in preterm neonates is associated with LOS, although the microbiome was reported to be highly variable in time (44, 45). Moreover, the causative pathogen retrieved in blood culture is often the most dominant species present in the gut microbiota. A recent prospective study by El Manouni et al. (46) showed that the causative pathogen could already be detected 3 days prior to LOS onset in fecal samples. These finding support the hypothesis of bacterial translocation, gut dysbiosis, and the occurrence of LOS.

**Figure 1:**
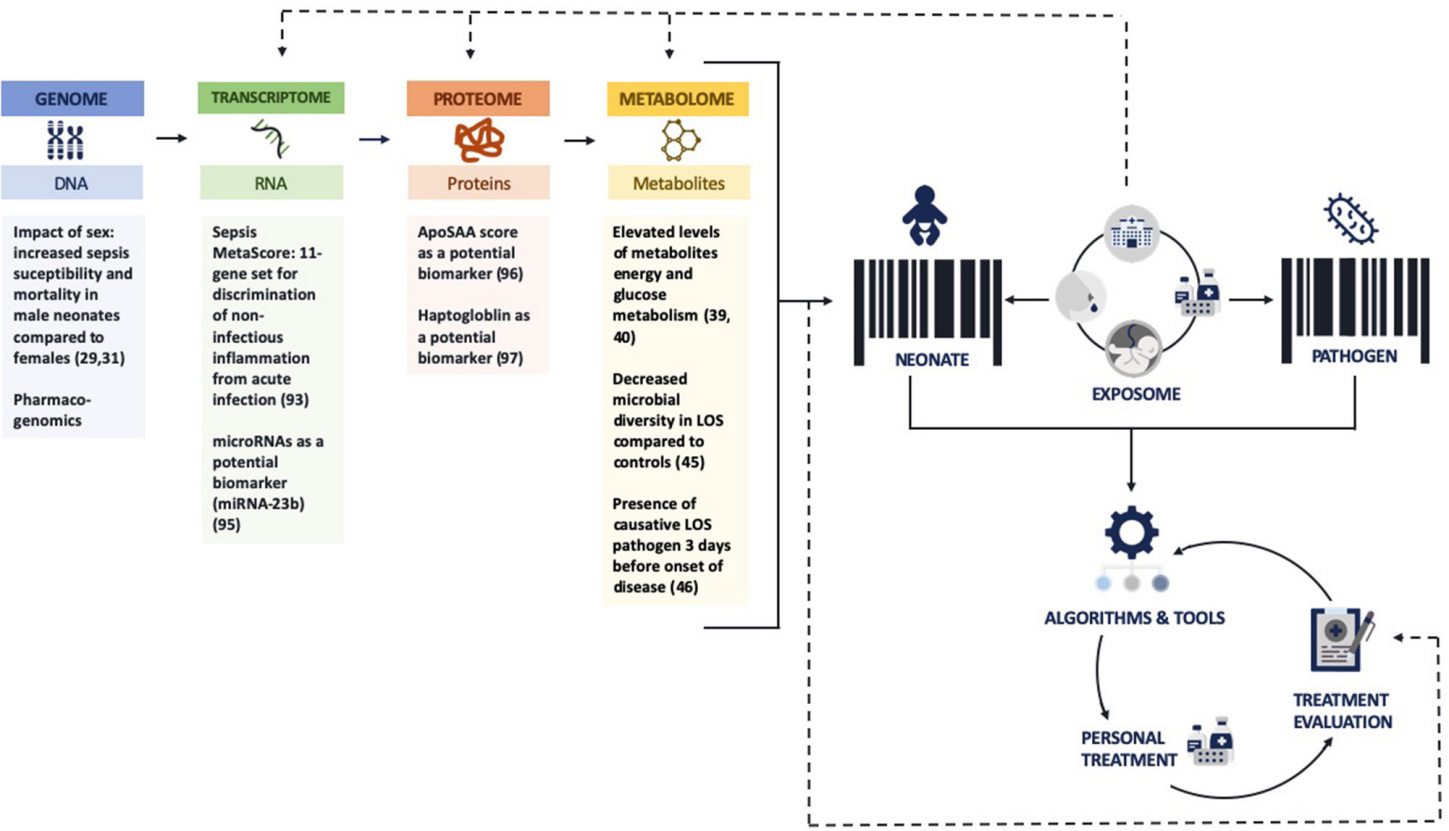
Concept of precision medicine and -omics techniques and its potential for neonatal bacterial infections. ApoSAA score, Serum Amyloid A (SAA) and Apolipoprotein (Apo)C2 score; LOS, late-onset sepsis. Figure (with all the icons) is created using https://www.flaticon.com/.

**Figure 2:**
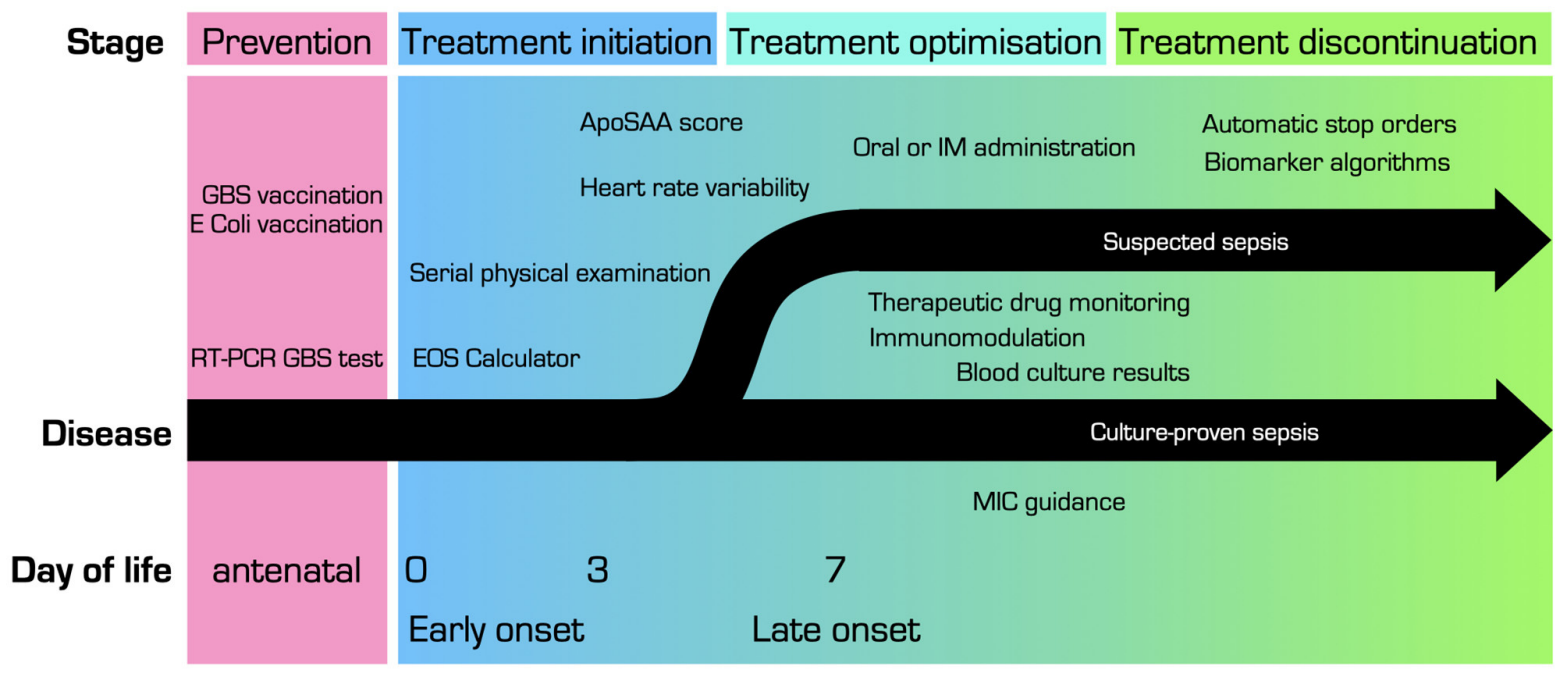
Overview of opportunities for precision medicine in treatment of (suspected) neonatal bacterial infection, at different stages of disease. ApoSAA score, Serum Amyloid A (SAA) and Apolipoprotein (Apo)C2 score; *E. coli, Escherichia coli*; EOS, early onset sepsis; GBS, Group B streptococcus; IM, intramuscular; MIC, minimal inhibitory concentration.

In the next paragraphs, we will elaborate on the current and future options to move toward a more stratified approach in the antibiotic management of proven and probable bacterial infection, focusing on late preterm and term neonates. These options include a wide variation of antibiotic stewardship programs and guidelines, clinical decision tools, pharmacological advances, biomarkers, and prevention strategies. We separate these options for each stage of decision-making that can be personalized: prevention, treatment initiation, treatment modality and optimization, and treatment duration. Figure 1 shows the concept of precision medicine and the use of -omics techniques with some recent findings relevant for neonatal bacterial infections. Figure 2 illustrates opportunities for precision medicine in neonatal bacterial infections during different phases of disease management. Finally, Table 1 presents a research agenda toward more precise medicine for neonatal bacterial infections.

**Table 1:** Current and future opportunities for precision medicine in neonatal bacterial infection management, with potential improvements, challenges, and specific research agenda items.

**Area of opportunity**	**Potential improvement**	**Main challenges**	**Research agenda**
**Prevention**			
Intrapartum antibiotics	Reduced incidence of neonatal sepsis	Appropriate and timely indication	RT-PCR implementation
Vaccination	Reduced incidence of neonatal sepsis	Achieving effective antibody levels	Phase II/III trials
**Treatment initiation**			
EOS calculator	Reduced overtreatment	Local implementation and evaluation	Cluster-randomized trials; integration in electronic healthcare systems
Serial physical examination	Reduced overtreatment; early sepsis identification	Few large studies; labor-intensive; lack of uniform practice	Development and testing of unified approach in large studies
Heart rate variability	Early sepsis identification; reduced mortality/morbidity	Very few validation studies; not validated for late preterm/term neonates	Validation studies, particularly for late preterm/term neonates
“Omics”	Improved diagnostics	Lack of validation; integration of systems biology into clinic	Validation studies of promising omics data; development of point-of-care biomarkers derived from omics data; studies focused on clinical decision-making
Computational power (machine learning)	Better identification of neonatal sepsis	Data collection and processing; validating models	Improving digital infrastructure; validation and implementation studies
**Treatment optimization**			
Oral administration	Less invasive treatment	Few data regarding safety/efficacy	Randomized trials for oral vs. intravenous treatment
IM administration	Availability in low-resource settings or in absence of intravenous access	Reducing pain; pharmacokinetic/pharmacodynamic uncertainties	Randomized trials for IM vs. intravenous treatment
Immunomodulation	Improved treatment efficacy: less mortality/morbidity	Limited knowledge on mechanism and efficacy	Randomized clinical trials
Therapeutic drug monitoring/model-informed precision dosing	Optimal pharmacological effect for individual	Lack of reliable/validated models	Model development and prospective validation
MIC guidance	Effective treatment	Lack of PK/PD data for neonates	PK/PD studies for (preterm) neonates
**Treatment duration**			
Automatic stop orders	Reducing overtreatment	Changing clinical paradigm	Quality improvement initiatives
Biomarker algorithms	Reducing overtreatment; better identification of sepsis	Limited or variable reference limits for biomarkers; limited sensitivity	Studies combining clinical parameters and multiple biomarkers; machine learning approaches
Blood cultures	Reducing overtreatment	Obtaining adequate volume; real-time blood culture reporting	Studies reporting time-to-positivity, Studies researching blood volume sensitivity; studies evaluating additional detection techniques
**General**			
Neonatal sepsis definition	Reliable and clinically relevant diagnosis	Defining criteria for organ dysfunction; defining long-term outcomes	Systematic reviews on organ dysfunction
Researching understudied populations	Improvements for relatively large population	Low sepsis incidence	Large cohort studies in late preterm and term populations

## Prevention

### Current Strategies

Mother-to-child transmission is considered to be the main route of transmission for EOS. In the presence of risk factors such as premature rupture of membranes (PROM) and signs of sepsis before birth, broad spectrum antibiotics are usually administered to the mother. To date, intrapartum prophylactic antibiotic therapy is only used in mothers with risk factors for GBS and has showed to be an effective way to decrease the transmission and incidence of GBS by 50–80% (47). However, several components and effects of this strategy are under debate, especially in the context of late preterm and term pregnancies.

First, considerable variation exists in maternal GBS colonization testing strategies (21). For instance, the American Academy of Pediatrics recommends universal antenatal GBS colonization testing in all pregnant women, and the use of intrapartum antibiotic prophylaxis in case of GBS colonization (48). In contrast, countries like the United Kingdom, Switzerland, and the Netherlands, opt for a risk-based management and only screen and treat high risk pregnancies (49). A disadvantage of the risk-based approach can be that cultures may be obtained too late to allow timely prophylaxis, whereas universal screening in the third trimester may not reflect the actual colonization status at birth. Second, the evidence with regard to optimal dose and timing of intrapartum antibiotics is evolving; it appears that the duration prior to birth is less important than previously thought (50). Third, there are concerns and uncertainties about potential adverse effects of intrapartum antibiotics on the neonate, such as perturbations of the developing microbiome (51, 52). A promising development for precision medicine in preventing neonatal EOS has been the application of point-of-care molecular testing for GBS colonization (53), which is likely to allow for quick and reliable qualification of maternal GBS colonization status and thereby facilitating more precise prophylaxis on admission. Validation and confirmation studies evaluating timing and dosage of intrapartum antibiotic prophylaxis as well workflows involving molecular testing are necessary to further improve this prevention strategy and minimize any of its adverse effects.

### Future Opportunities

#### Vaccination

Vaccination is traditionally used as a preventive measure targeting the general population and applied at a universal scale. However, there is increasing interest on the use of “*precise vaccination*,” targeting specific subpopulations and tailoring vaccination on a more individual level, taking into account factors such as age, sex, and disease susceptibility. Adjusting formulation, dosage and timing to patient factors could help maximize the effects of vaccination while reducing the risks (54). In the specific setting of neonates, immunization through vaccination of subpopulations of pregnant women could be an elegant manner to protect the neonate against invasive infections in the first 3 months of life, including bacterial infections. Vaccine-specific IgG can be transferred across the placenta during late-second to third trimester and provides a time window for effective and safe vaccination (55). To illustrate feasibility and relevance of this approach, safety, and efficacy has been documented for maternal pertussis vaccination which resulted in higher antibody concentrations in newborns in the first 3 months of life and a maternal vaccination program has already been implemented in several countries (56, 57). Similar efforts are ongoing for GBS and *E. coli*.

Several phase I/II trials in non-pregnant and pregnant women have evaluated the safety and tolerability of a multivalent GBS vaccine. Studies have shown the vaccine to be safe and did not report related major adverse events in vaccinated women. GBS-specific antibody responses were significantly higher among vaccinated women compared to controls (58). A phase II study reported that vaccination reduced the vaginal and rectal GBS colonization in healthy non-pregnant women (59). Only a few studies evaluated safety for the fetus and no severe events have been reported in offspring. Women are vaccinated in their third trimester and therefore toxicity for the fetus is considered to be low (60). Unfortunately, this also means that vaccination would mainly be effective to prevent invasive GBS infection in late preterm and term neonates. Earlier vaccination, during the second trimester, would be needed to protect preterm neonates, although transplacental antibody transport is reduced before the third trimester, resulting in lower anti body concentrations in general following preterm birth (61). To our knowledge, there are no phase III trials currently ongoing but maternal immunization could be a potential additional strategy to further reduce the burden of GBS infection (55, 62).

As *E. coli* infections are associated with substantial morbidity and mortality among newborns it would be tempting to evaluate whether maternal immunization, as discussed for GBS, could be beneficial for *E. coli* infections as well. Till date, trials on *E. coli* vaccination mainly focus on vaccination against Enterotoxigenic *E. coli* (ETEC), which is associated with childhood and travelers' bacterial diarrhea with a high mortality (63). A rodent study examined the use of Outer membrane protein A (OmpA) based vaccine for *E. coli*. Gu et al. were able to generate an artificial protein (OmpAVac) which was subsequently injected in adult and neonatal mice. They report an increased specific antibody response and better survival in immunized mice, including neonatal mice (64). A first in human phase 1b randomized clinical trial evaluated the safety and immunogenicity of a bioconjugate vaccine containing the O-antigens of four *E. coli* serotypes (ExPEC4V) in healthy, non-pregnant women with recurrent urinary tract infections. Authors reported no vaccine related adverse events and elevated antibody responses were detected against all four serotypes compared to placebo (65). These studies highlight the potential for further research on *E. coli* vaccination against invasive infection, with special focus on maternal immunization and neonatal protection.

## Treatment Initiation

### Current Strategies

National guidelines, taking into account both maternal and neonatal risk factors and the clinical condition of the patient, have been developed to provide support for recognition and optimization of diagnosis and treatment of neonatal infections, especially for EOS (11, 21). Much less guidance is present for suspected LOS; therapy is usually initiated when clinical signs are present. Overall, guidelines for EOS on treatment initiation show similarities: antimicrobial treatment is often initiated based on the presence of risk factors or non-specific clinical symptoms. With regard to treatment initiation, some guidelines, such as the Swiss guideline, recommend clinical observation with monitoring of vital signs every 4 h for a period of 48 h in asymptomatic neonates with risk factors while other guidelines recommend treatment initiation even in the presence of risk factors only (21, 48, 66).

Due to low specificity of mentioned risk-factors as well as clinical signs at onset of possible bacterial infection, a quest for biomarkers to assist in the decision-making regarding initiation of antibiotics has been ongoing for years. A long list of hematologic parameters, interleukins, endothelial molecules, and various other biomarkers have been or are currently being evaluated for the early detection of neonatal sepsis (67, 68). There is, in general, consensus that classic and well-researched biomarkers such as the complete blood count and C-reactive protein (CRP) are insufficient to guide the initial decision on antibiotic initiation (69). For EOS, this is largely due to physiological fluctuation of thrombocytes, leukocytes, and CRP after birth (11, 67). For LOS, low specificity as well as a delay of hours between infection and rise of biomarker levels currently limits the usability of biomarkers at moment of infection suspicion (67). Further research is needed to analyze if biomarkers, especially those reflecting inflammation early in the sepsis course, such as interleukin-6, may be used to inform precise treatment decisions beyond the start of antibiotics, such as start of adjuvant therapies, transferal to higher levels of care, or inotropic support (70).

Several developments may allow transition to a more precise risk assessment without the need for biomarker analysis as a basis for the decision to start antibiotics or not. The neonatal EOS Calculator (kp.org/eoscalc) has been developed based on a dataset of over 600,000 term and late-preterm neonates. It provides an individual quantitative risk estimate calculated from five quantitative objective risk factors at birth (exact gestational age, highest intrapartum maternal temperature, duration of ruptured membranes, and maternal GBS colonization status), and an assessment of the neonate based on objective clinical parameters (71, 72). Although several characteristics of the EOS Calculator affect the accuracy of the individual risk estimates (73), it has proven markedly useful for risk stratification. Studies have shown that its implementation is associated with a marked reduction (relative risk reduction 44%) in neonates receiving empiric antibiotics, without occurrence of adverse effects such as increases in sepsis incidence or worse clinical outcome (74, 75). Although the EOS Calculator does provide an individual risk estimate, such risk stratification remains imperfect, meaning that clinical vigilance remains mandatory even for low-risk neonates (73, 76).

An approach completely depending on the clinical vigilance is the use of serial clinical observations in term neonates. It encompasses structured and repetitive examinations of selected or all newborns by a skilled and trained nurse or physician, for the first 24–48 h postpartum (77, 78). It deliberately restricts antibiotic treatment initiation to clearly symptomatic neonates, and can reduce the rate of antibiotic treated neonates for suspected EOS to as low as 1.3% (compared to 2.9% pre-implementation) (79). Protocols for serial clinical observations differ greatly but require intensive individual medical assessment, such as hourly physical examinations. This approach has, until now, only been evaluated in a few, mostly well-staffed settings (79, 80). As a result, safety data are still limited, and the approach may not easily be implemented in settings unable to provide repeatedly assessments by clinical professionals.

For neonates admitted beyond the 1st days of life, clinicians may face similar clinical decision dilemmas if signs of a bacterial infection become present but may be explained by other factors or diagnoses. This may lead to both unnecessary treatment of uninfected neonates, as well as delayed treatment initiation in sick neonates.

### Future Opportunities

The use of physiomics such as heart rate variability (HRV) can possibly contribute to earlier infection recognition. The autonomic nervous system plays an important role in the maintenance of body homeostasis and regulates, among other processes, the beat-to-beat variability of the heartbeat (81). HRV in turn, is linked to other vital signs such as respiration and blood pressure. Sepsis, especially the presence of endotoxins, can induce autonomic dysfunction which leads to a decreased HRV, which in turn has been associated with a higher disease severity and mortality among septic patients (82, 83). Moreover, HRV is commonly used to monitor fetal condition during labor using cardiotocography and a decreased HRV can be used as a predictor for fetal distress such as intra-uterine infections (84, 85).

In preterm neonates, a decrease in HRV in combination with the presence of transient decelerations has showed to be an early predictor for sepsis and has consequently led to the development of a heart rate characteristics (HRC) monitor. This monitor can be used to identify patients at risk for developing LOS in the next 24 h, allowing timely initiation of antibiotic therapy (86). A large randomized clinical trial showed a significant reduction in mortality among (22% relative reduction from 10.2 to 8.1%) preterm neonates in whom HRC scores were displayed to the treating physician. Authors did however report an increase in sepsis workups and days on antibiotics (87). A retrospective study in which scores were available twice daily reported a limited usability of the HRC score as many elevated scores were not related to a LOS episode (poor specificity) (88). It is known that non-infectious conditions such as medication (dexamethasone, paralytics, and anesthetics), surgery, initiation of mechanical ventilation, and bronchopulmonary dysplasia can also influence the HRC score (86, 89, 90). Although the use of HRV does not seem to be limited to preterm neonates, the HRC monitor has not yet been validated in late preterm and term neonates, nor for sepsis episodes that occur in the first 72 h of life (88, 91).

The Sepsis MetaScore (SMS) is diagnostic test aiming to diagnose sepsis based on gene expression. The SMS has been developed using a multicohort analysis and consists of an 11-gene set that can discriminate non-infectious inflammation from acute infection. It has been validated in several transcriptomic cohorts of adults and pediatric patients (92). It has recently been validated in three genome-wide expression based neonatal cohorts and has a high diagnostic accuracy for the discrimination of non-septic from septic neonates. Moreover, when combined with “traditional” biomarkers (white blood count, CRP, and neutrophil count) it improved the diagnostic accuracy of all three biomarkers, mainly because it led to a rule-out of sepsis (specificity) among low-risk patients. Moreover, and very relevant for the late preterm and term population, the SMS is capable of distinguishing neonates with suspected sepsis from those with confirmed sepsis (AUC: 0.90). Although the SMS needs to be further evaluated in prospective studies, it underscores the potential for transcriptomics to guide treatment (93).

Micro-ribonucleic acids (miRNAs) are involved in different cell processes such as cell signaling and immune activity and could therefore serve as potential biomarker for the diagnosis of sepsis. Specifically, miRNA-23b has been associated with the regulation of innate immunity and its expression is related to inflammation. In adults with sepsis, lower levels of miRNA-23b were associated with sepsis and mortality among septic patients. Within the sepsis group, lower levels were seen among non-survivors (94). This illustrates its usability for both diagnosis and severity grading of sepsis. It has therefore been evaluated in a small cohort of preterm and term neonates with EOS and LOS (95). Reduced miRNA-23b expression compared to controls was seen in both preterm and term neonates who died from EOS. Among EOS survivors, miRNA-23b expression was higher compared to controls, thus expression seems to correlate with sepsis progression. In LOS cases, miRNA-23b expression was lower in all septic neonates (both survivors and deaths) compared to non-septic controls.

Proteomic studies led to the development of the ApoSAA score, which combines serum amyloid A (SAA) and Apolipoprotein (Apo)C2. A case control study among preterm neonates showed that the ApoSAA score can differentiate non-septic infants from LOS or necrotizing enterocolitis cases (96).

Another proteomic study by Buhimschi et al. (97) revealed haptoglobin and haptoglobin-related protein immunoreactivity as a potential additional biomarker for EOS. In this study, cord blood of presumed and proven EOS patients was profiled, identifying significantly elevated levels in neonates with EOS. Further research is needed to evaluate the usability of miRNAs, the ApoSAA and haptoglobin as new biomarkers for neonatal sepsis.

## Treatment Optimization

Antimicrobial stewardship programs have been developed aiming to optimize clinical outcomes while reducing the negative consequences of antimicrobial use (17). These contribute to further tailoring of antibiotic therapy and have shown to be beneficial in the reduction of unnecessary antibiotic use and prevention of antimicrobial resistance (19). Principles of these programs are *appropriate selection, appropriate administration*, and *timely de-escalation* (20).

### Current Strategies

The predominantly causative pathogens are the main determinant for correct choice of antibiotic therapy. For both EOS and LOS, it holds true that, at moment of infection suspicion, the possible causative pathogen of the infection and the antibiotic susceptibility test are not yet known. For EOS, empiric therapy usually consists of a combination of a penicillin with an aminoglycoside. For LOS a wider variety of combinations is used (98). Importantly, the choice of the antibiotic regimen highly depends on causative pathogens and antimicrobial resistance rates which differ substantially throughout Europe and worldwide (99). In the absence of strong evidence and in part related to differences in product availability and preferences, variability in daily practice between units, especially for LOS, is extensive (98).

After identification of a pathogen, its susceptibility for a specific antibiotic is defined by the minimal inhibitory concentration (MIC) which is the lowest antibiotic concentration needed to prevent further replication of the pathogen and is thus of importance for the determination of the dosing regimen (100). In order to facilitate precise antibiotic treatment, several pharmacological components should be considered. Drug dosing regimens have commonly been extrapolated from adult studies and practices, thus the majority of drugs prescribed to neonates are off label (101). However, neonates differ substantially from older children or adults, thereby influencing the pharmacokinetics (PK; what the body does to the drug) and pharmacodynamics (PD; pharmacological response of the body to the drug) of a drug. Simplistic extrapolation from adults and children to neonates could lead to under- (compromising efficacy) or overexposure (risking toxicity) (102). Consequently, drug dosing regimens should ideally be based on integrated knowledge concerning the disease to be treated, the physiological characteristics of the neonate, and the PK/PD of a given drug.

#### Pharmacokinetics/Pharmacodynamics of Antibiotics

Efficacy of antibiotics strongly depends on the mode of action of the chosen class. This can be time-dependent killing (Time > MIC; beta-lactam antibiotics), concentration-dependent killing [maximum concentration (Cmax)/MIC; aminoglycosides] or combined time- and concentration-dependent killing [area under the curve (AUC)/MIC; vancomycin] (91). Besides these targeted effects, antibiotic exposure also results in off target effects like alterations in the gut microbiome. Intriguingly, these alterations themselves can also alter enteral drug and first pass metabolism (103).

PK compromises the process of *absorption, distribution, metabolism*, and *elimination* and both maturational and non-maturational covariates can impact a dosing regimen (*get the dose to target*). The distribution of antibiotics is driven by maturational differences in body composition (water %), by presence of disease or by treatment modalities, like extra-corporeal membrane oxygenation (104). Plasma protein concentration and binding capacity may also be of relevance for some protein bound antibiotics, like cefazolin or vancomycin. This because the fraction of time during which the free, unbound (%*f* T), antibiotic concentration is above a given MIC (%*f* T > MIC) is the efficacy target (105, 106). Subsequent elimination of antibiotics is almost exclusively by renal elimination, and to a smaller degree through metabolism or biliary elimination. The main factors involved in the development of renal function are GA, postnatal age and birth weight (107). This results in rather complex dosing regimens within the neonatal population, as reflected in different recent reviews on this topic (108, 109). Only for specific antibiotics, drug metabolism by cytochrome P450 (CYP) or glucuronidation) is involved in its clearance. Consequently, the clearance of erythromycin (CYP3A), clindamycin (CYP3A), or chloramphenicol (glucuronidation) clearance is driven by the maturational activity of these enzymes, further affected by non-maturational changes like genetic polymorphisms or disease characteristics (e.g., inflammation affects CYP3A activity) (110).

### Future Opportunities

#### Therapeutic Drug Monitoring and Model Informed Precision Dosing

Therapeutic drug monitoring (TDM) is used to optimize antibiotic dosing and is especially of interest in case of a narrow therapeutic window. Moreover, it can be informative for drugs that show a large interpatient variability, as serum concentration predictions can be difficult. TDM has historically been developed to prevent toxicity. However, nowadays it is also used to guide therapy. It can be applied in drugs for which a correlation is present between serum concentrations and the pharmacological effect of the drug, thus the concentration in the target tissue. Moreover, the pharmacological effect should not be easily measurable through less invasive methods and a quantification method should exist (111).

TDM is not commonly used for dosing of beta-lactam antibiotics. They have a broad therapeutic window and are, in general, perceived to be not very toxic. However, given the increase in antimicrobial resistance and reported increase in MIC of certain pathogens, and thus narrowing of the therapeutic window in time, it could be beneficial to use TDM for other antibiotics to confirm target attainment. Moreover, most of beta-lactam antibiotics are renally cleared, and thus as previously discussed, the concentration-time profiles can be influenced by maturational and non-maturational covariates (107).

Finally, the most appropriate pharmacokinetic target remains a point of discussion. In neonates, who are perceived as relatively immunocompromised, a T > MIC of at least 40–50% of the dosing interval is recommended (112). However, on the adult ICU, targets for critically ill patients range from 100% T > MIC up to 100% T > 4 × MIC so one could question whether 40–50% is enough in neonates (113). It is important to notice that the used concentration is the free unbound concentration, which is the antimicrobial active part of the drug. This fraction varies between neonates and adults and most centers measure total concentrations, therefore a correction should be applied when interpreting the concentrations (100, 113). The relevance of this free fraction and protein binding has recently been explored for the free vancomycin AUC target to consider in neonates (105). The higher unbound vancomycin fraction in neonates can result in a lower dosing regimen.

Model-informed precision dosing (MIPD) is an obvious next step for TDM and has recently gained more attention as it may serve as a powerful tool to help individualize dosing. MIPD is a next generation dosing paradigm in which mathematical models, in combination with individually measured patient characteristics (e.g., drug concentration, genotype, organ function) and disease characteristics (e.g., pathogen susceptibility), are used to calculate the optimal dose (114). Bedside integration of combined data on exposure and effect would allow a quick/real-time individualization of dosing and target attainment (115). For neonates, this has yet been evaluated for amikacin and vancomycin. A prospective study on amikacin evaluated a model-based dosing regimen in neonates reporting optimized concentrations in almost all neonates with use of the model (116). A retrospective study on vancomycin also reported improved target attainment in neonates when using a model-based dosing approach (117).

#### Routes of Administration

Antibiotics are most commonly administered intravenously to hospitalized newborns in the 1st weeks of life. It allows precise and direct drug disposition into the circulation, but it requires intravenous access. Moreover, dissolving all drugs and flushing the lines in between administrations may contribute to a fluid overload (118). Late preterm and term neonates, in general, are not dependent on central access for feeds and fluid and receive the venous access solely for the administration of antibiotic therapy. As intravenous therapy is generally only provided in hospital, this leads to prolonged hospitalization of an otherwise relatively healthy newborn.

Intramuscular administration has been evaluated in several large trials, especially in low- and middle-income countries, as an alternative in case referral to a hospital is not possible and can be an effective alternative when intravenous administration is not possible (119). A study showed that, although parents recognize intramuscular administration to be more painful, they may still prefer this modality as to their opinion, it allows better bonding and breastfeeding compared to intravenous administration (120).

Oral administration of antibiotics is not commonly performed in newborns in the 1st months of life because of uncertainties on absorption, bio-availability and target exposure. However, several small pharmacokinetic studies have evaluated the use of oral antibiotics in neonatal infections and although absorption is slower compared to that of older children and adults and inter-individual variation is seen, target levels can be reached following oral administration (121). With regard to efficacy, several large trials have evaluated the use of an oral regimen in low- and middle-income countries. No increase in mortality or adverse outcome was reported. Unfortunately, results are not applicable to a high-income setting as pathogen distribution and the availability of diagnostics differs substantially (119, 121). A randomized clinical trial evaluating the effectiveness and safety of intravenous-to-oral antibiotic switch therapy in neonates with a probable bacterial infection is currently ongoing and results are expected by the end of 2021 (NCT03247920) (122).

#### Add-On Therapies and Immunomodulation

Antibiotic therapy solely targets the causing pathogen but does not target the host' immune system and the subsequent inflammatory responses (123). Immunomodulation, targeting specific cellular and molecular processes involved in the development of neonatal sepsis, could be a very promising add-on therapy. Unfortunately, many trials have failed to show efficacy in neonatal sepsis (124, 125).

The transplacental transport of immunoglobulin G (IgG) occurs for the greater part, in the third trimester (GA > 32 weeks), thus very preterm neonates are considered immunoglobulin deficient. Therefore, several clinical trials have evaluated the use of iv immunoglobulins (IVIG), both for prevention and treatment of infection in neonates. A recently updated Cochrane Review evaluating the additional benefit of IVIG in neonates (<28 days of age) with suspected or proven bacterial infection reported no significant difference in mortality between IVIG treated patients and placebo (126). When only looking at studies in preterm neonates (GA < 37 weeks) at risk for LOS, a small, but significant reduction in the incidence of sepsis was reported (3%; number needed to treat: 33). However, no significant reduction in mortality from infection, necrotizing enterocolitis, bronchopulmonary dysplasia or intraventricular hemorrhage was seen. With regard to safety, no major adverse reactions following iv administration of IVIG were reported (127). As a clear benefit of IVIG is lacking, it may be hypothesized that pathogen-specific immunoglobulins could contribute to sepsis management. However, a clinical trial evaluating the use of anti-staphylococcal human immunoglobulins showed no significant differences in *S. aureus* and CoNS sepsis rates, nor in mortality rates, between treatment and placebo (128).

Pentoxifylline (PTX) could be a promising drug; it is a vaso-active drug, originally developed for the treatment of claudicatio intermittens in adults. It has anti-inflammatory effects that influence cytokine production possible attenuating the hyper inflammatory response associated with neonatal sepsis. Moreover, it also influences the microcirculation, which is often impaired in neonatal sepsis (129, 130). A large international randomized placebo-controlled trial in currently ongoing in which the survival of preterm neonates (GA < 29 weeks) following additional treatment with PTX when LOS is suspected, is evaluated (ACTRN12616000405415). Next, and very relevant as PK studies on the use of PTX for neonatal sepsis are currently lacking, a single center dose optimization study is currently ongoing in which the optimal dose is studied (NCT04152980).

## Treatment Duration

### Current Strategies

Treatment duration depends on several aspects and is ideally based on the causative pathogen. However, in many cases, cultures remain negative and therapy is continued because laboratory or clinical signs of infection remain present. In that case, guidelines recommend continuation of antibiotic therapy for 5–7 days (49). For proven bacterial infections, there appears to be little evidence for current treatment durations. For now, the questionable “magic numbers” for treatment duration are 7, 10, 14, and 21 days, depending on the cultured microorganisms. A trial for radiologically proven neonatal pneumonia (without bacteremia) did not indicate a difference in treatment success between a 4 and 7-day treatment (131). For uncomplicated GBS infections, intravenous treatment for 10 days is recommended. A retrospective analysis showed however that, in some cases, a shorter course (≤8 days) is prescribed. Patients receiving a short IV course were older compared to patients receiving prolonged IV therapy. Recurrence rates were not higher in the short IV therapy group (132). A systematic review on the evidence of short vs. long duration of antibiotic treatment for neonatal bacterial infections is currently underway (133).

### Future Opportunities

In contrast to tools providing individual risk assessments to guide the initiation of antibiotics, only a few tools allow truly individualized decision making with regard to treatment duration in neonatal infections. These decisions therefore mostly rely on clinical judgment, but data from recent studies provide opportunities for improving tailored decision making. As blood cultures remain the best proxy for a definitive diagnosis (134), average time-to-positivity can be an important variable when considering (dis)continuation of treatment. Recent data show this time-to-positivity is <36 h for at least 94% of positive blood cultures obtained for suspected EOS, and <24 h for at least 68% of those (135, 136). Depending on *a priori* risk, clinical course, and infection parameters, this may facilitate discontinuation of antibiotics at these time points. Molecular techniques using DNA amplification are promising as they can detect bacterial, viral, and fungal material and have a shorter turnaround time of on average 6 h in comparison to culture-based methods. Unfortunately, at this moment, these techniques can only be used as add-on diagnostics as contaminant detection and negative results in culture positive infections have been reported. Moreover, pathogen susceptibility testing, crucial for targeted therapy, is not possible using these techniques (134).

Automatic stop orders enforce proactive decisions on continuation, which may induce more personalized decision-making, and can be highly effective in reducing unnecessary continuation of antibiotic treatment (137, 138). Such personalized decisions may be further improved if guided by combining risk stratification and age-dependent reference values for biomarkers such as procalcitonin (PCT) and or CRP (139–141). Serial low CRP values, normal PCT values, or the combination of CRP and PCT can support discontinuation of antibiotics (142). The use of PCT as guidance for antibiotic treatment in late preterm and term neonates has been evaluated in a large clinical trial (NeoPInS study) (143). The study showed that PCT-guided decision making can reduce the duration of antibiotic therapy (55 h intervention group vs. 65 h control group) in neonates treated for suspected EOS (139). The algorithm has recently made available as a mobile application (NeoPInS app; Apple app store/Android) and can be used in daily clinic as support tool in late preterm and term neonates with suspected EOS.

## A Research Agenda Toward Precision Medicine

Millions of late-preterm and term neonates are born each year and are potentially at risk for bacterial infections. A “one size fits all approach” is inappropriate for this population, which is susceptible to consequences of both under- and overtreatment. The relatively low incidence of sepsis in this group is a key research challenge and calls for concerted and widespread collaborations. Aiming for a future in which precision medicine mitigates the risks of these consequences using a tailored approach, we propose the following research agenda. Specific research opportunities for this agenda are listed in Table 1.

### Step 1: Reaching Consensus Definitions

As mentioned before, a consensus definition of neonatal sepsis is critically lacking. The lack of agreement on the definition of what consists a “sepsis case” not only hampers clinical diagnosis, but also hinders research aiming to provide tailored approaches. The myriad of terms such as “culture-negative sepsis,” “probable sepsis,” or “clinical sepsis” makes comparing studies and their outcomes difficult, renders implement research findings into clinical workflow challenging, and sustains discussions that prevent research progress. Efforts that provide objective and measurable criteria to define a case of neonatal sepsis and/or define the need for (sustained) antibiotic treatment constitute a first step in progressing precision medicine, because such criteria will be highly beneficial to the development and implementation and evaluation of precision medicine tools as described in this paper.

### Step 2: Implementing Current Opportunities

In spite of the limitations of hitherto used proxy definitions, an array of current opportunities to tailor medicine for neonatal bacterial infections are readily available for clinical implementation today. For example, the EOS calculator is endorsed by academic societies and widely being implemented (22, 144). Likewise, automatic stopping orders are facilitated by most of today's electronic health care information systems. Interdisciplinary efforts are envisioned to make MIPD widely available in clinical practice (145). Despite their imperfections, careful implementation of currently available tools presents a large first step toward precision medicine and can directly impacts today's patients.

### Step 3: Addressing the Understudied Population

Relatively few clinical trials involving precision medicine have focused on late preterm or term neonates. Consequently, several of the tools developed for preterm neonates have not yet been evaluated in term neonates. Although preterm neonates are more at risk for severe disease and bad outcomes, the absolute numbers of late preterm and term neonates render these an understudied group for precision medicine, with large potential for reductions of antibiotic use, hospitalization rates, economic costs and improvements of quality of life. Validation and development of precision medicine tools should therefore regain priority, recognizing key differences from preterm neonates. Examples of these include differences in pathogen distribution, maturation-specific pharmacokinetics (volume of distribution, renal excretion), and specific immune responses (such as mild disease course of CoNS infections).

### Step 4: Leveraging New Research Fields

#### Omics

The use of multi-omics is an exciting development in the field of neonatal bacterial infections. It has the potential to reveal a patients' unique disease signatures in response to a pathogen, allowing tailored therapy and disease management. However, additional clinical studies on the development and integration of these -omics derived biomarkers into daily clinic are required.

#### Computational Power

Medical data analysis and decision-making can be done by humans without technology, by humans assisted by technology, or completely machine-guided. Today, this decision-making spectrum contains a myriad of possibilities including straightforward decision aiding tools based on traditional statistics, up to and beyond complex variations of machine learning and artificial intelligence (146). Improvements in computational power of recent decades allow for analysis of massive amounts of data, and leveraging this power is projected to fundamentally alter medical practice (147). Machine learning models to improve precision in neonatal sepsis management are being developed, but it will take adaptation of the medical electronic infrastructure, evaluation cycles, and scientific research to allow the promises of true clinical impact to become reality (147–149).

## Conclusions

The global burden of suspected neonatal infections remains high in both preterm and term neonates. This results in a high antibiotic exposure in the first weeks of life. The heterogeneity of disease, together with increasing evidence with regard to the negative effects of antimicrobials and emerging resistance rates, ask for a holistic disease approach and improved treatment strategies. Precision medicine is a promising development involving improved stratification of neonates at every stage of management, thereby facilitating precise balancing of under- and overtreatment.

Key challenges in finding that balance consist of selective but timely administration of antibiotics to those who need treatment, and discontinuation or de-escalation of antibiotic therapy when possible. Fortunately, continuous effort on the development of prediction tools has shown to be beneficial and led to a reduction of antibiotic prescriptions. Further research on potential biomarkers, using omics, could lead to a combined risk stratification tool. Moreover, in case of a true infection, those tools would give insight in the patient specific immune signatures triggered by the interaction of the host and the causative pathogen and possibly even predict disease severity, thereby allowing early, targeted initiation of supplemental therapy next to antibiotics.

In addition, therapy should be safe and effective and one should be well aware of the negative side-effects of treatment such as microbiome perturbations. As the host-pathogen interaction (“disease signatures”) is unique, treatment should be guided using biomarkers and TDM instead of being standardized for all patients. Add-on therapies such as immune modulation should be considered based on these disease signatures and should be administered timely and only when indicated.

To achieve these objectives in a field with scattered but promising developments, there is a need for focused and concerted research efforts. For this, we propose a research agenda (Table 1) with distinct implementation and development opportunities toward a reality of precision medicine in neonatal bacterial infections. This agenda is certainly not exhaustive, but may serve as guidance in upcoming research efforts and can be adapted with complementary and promising developments.

In conclusion, the field of precision medicine is an exciting development offering many opportunities for better management of neonatal bacterial infections. Some tools, especially decision-making tools and algorithms are readily available for implementation, whereas other tools such as therapeutic drug monitoring and the use of *omics* still require further development or validation. Research may focus on the late preterm and term population and how they respond to infection early in life as this group remains relatively understudied. Finally, in the near future, advances in data science and analysis are likely accelerate developments in precision medicine for neonatal bacterial infections.

## Author Contributions

FK and NA wrote the first version of the manuscript. KA and RK supervised the writing of the first draft. GT-S, AR, and IR critically reviewed and rewrote the manuscript. All authors approved the final manuscript as submitted and agree to be accountable for all aspects of the work.

## Conflict of Interest

The authors declare that the research was conducted in the absence of any commercial or financial relationships that could be construed as a potential conflict of interest.

## Abbreviations

EOS: early-onset sepsis
GA: gestational age
*E. coli*: *Escherichia* coli
GBS: group B *Streptococcus*
LOS: late-onset sepsis
CoNS: coagulase-negative staphylococci
NICU: neonatal intensive care unit
CRP: C-reactive protein
HRV: heart rate variability
HRC: heart rate characteristics
MIC: minimal inhibitory concentration
MIPD: model-informed precision dosing
PK: pharmacokinetics
PD: pharmacodynamics
Cmax: maximum concentration
AUC: area under the curve
%*f* T, percentage of time fraction of the free: unbound concentration
TDM: therapeutic drug monitoring
IgG: immunoglobulin G
IVIG: iv immunoglobulins
PTX: pentoxifylline
PCT: procalcitonin
RNA: ribonucleid acid
MMP8: metalloproteinase-8
TNF-α: tumor necrosis factor-alpha
PROM: premature rupture of membranes
ETEC: enterotoxigenic *e.coli*
SMS: Sepsis MetaScore.
